# P07-01 Prescription of adapted physical activity: knowledge and needs among general practitioners of Ile-de-France

**DOI:** 10.1093/eurpub/ckac095.101

**Published:** 2022-08-29

**Authors:** Alexis Astruc, Jean-Christophe Blanchard

**Affiliations:** 1 Department of General Practice - University Paris 13, Bobigny, France; 2 Ellasanté Health Center, Paris, France

**Keywords:** physical activity prescription, exercise, primary care, general practitioners, observational study

## Abstract

**Background:**

Physical inactivity is the fourth leading cause of death in the world. In France, the concept of medical prescription of physical activity (PA) adapted now appears in a law which specifies the central role of the family physician since December 2016. The needs of the general practitioners (GP) about prescription of PA has not been studied.

**Aim:**

To assess the knowledge and needs of GPs regarding the medical prescription of PA.

**Methods:**

This is a transversal observational epidemiological study of GPs working in the departments of Hauts-de-Seine (92) and Val-de-Marne (94) near Paris. A questionnaire was sent by mail or email. A descriptive statistical analysis was conducted to describe the main variables of interest. Then, a multivariate statistical analysis by logistic regression was conducted to look for independent factors of the feeling of competence in the prescription of adapted PA.

**Results:**

158 physicians were included. 72.2% of GPs rated their knowledge in PA as average to very poor. The initial training in PA is estimated as unsatisfactory by 84.2% of GPs. 81.6% haven't done a postgraduate additional training in PA. A specific skill in sports medicine, the individual practice of PA as well as the speaking about PA in more than 50% of consultations are significantly associated with a feeling of competence in PA prescription (*p* >0.01).

Among the measures favoring the prescription of PA, GPs told that they need information brochures to give to the patient (60.8%), a website of help to the prescription of PA which can be used in consultation (60.1%), or the organization of a specific training on the medical prescription of AP (50,6%).

**Conclusion:**

The lack of training seems to be the major obstacle to the generalization of prescription of PA. The organization of training or the creation of specific tools for the attending physician seems to be promising solutions.

